# Muscle fiber and fresh meat characteristics of white-striping chicken breasts, and its effects on palatability of sous-vide cooked meat

**DOI:** 10.1016/j.psj.2021.101177

**Published:** 2021-04-11

**Authors:** Boin Lee, Chun Ho Park, Changsu Kong, Young Soon Kim, Young Min Choi

**Affiliations:** ⁎ Department of Animal Sciences, Kyungpook National University, Sangju, South Korea; † Department of Integrated Biomedical and Life Sciences, Korea University, Seoul, South Korea; ‡ Department of Hotel and Food Service Culinary Art, Daejeon Health Institute of Technology, Daejeon, South Korea

**Keywords:** white-striping chicken, sous-vide, muscle fiber, meat quality, sensory quality

## Abstract

The aim of this study was to compare the histochemical and meat quality characteristics between the normal and white-striping (**WS**) *pectoralis major* muscles. Additionally, this study investigated the effects of oven cooking (**OV**) and sous-vide (**SV**) cooking methods on objective texture parameters and sensory quality characteristics of the normal and WS chicken breast meats. Results showed that the WS condition broilers had higher body and breast weights (*P* < 0.001), and a greater area of muscle fiber than the normal broilers (*P* < 0.001). The WS fresh fillets exhibited a lower preference of visual appearance compared to the normal fillets (*P* < 0.05), although no differences were detected in the characteristics of meat quality between the groups (*P* > 0.05). After cooking, there was greater cooking loss, Warner-Bratzler shear force, and texture parameter analysis-hardness values for breast fillets cooked by OV treatment at 180°C for reached core temperature to 71°C compared to the fillets cooked by SV treatment at 60°C for 2 h (*P* < 0.05), whereas the normal and WS groups were exhibited similar values within each cooking methods (*P* > 0.05). Regarding sensory quality characteristics, WS breast fillets cooked by SV (SV+WS) were rated as tenderer and juicier, and given a higher overall acceptability score compared to normal and WS fillets cooked by OV (*P* < 0.05). However, owing to a lesser developed flavor in SV+WS fillets, the panelists assigned a lower overall acceptability rating in these fillets compared to SV+Normal fillets (*P* < 0.05). Overall, the SV cooking can be an effective method for improving the sensory quality characteristics of WS and normal chicken breast.

## INTRODUCTION

The consumption per capita of poultry meat, especially chicken, has been increasing due to growing consumer interest in healthier diets (Petracci and Cavani, 2012). In response, the poultry industry has been focusing their production efforts on maximizing muscle development and growth (Barbut et al., 2008). Generally, broilers grown rapidly exhibited greater muscle weight, especially *pectoralis major* (**PM**) muscle, compared to broilers grown slowly, and rapid growth rate was associated with changes of muscle fiber characteristics before and after hatching (Choi et al., 2013; Petracci et al., 2015). Histochemical changes and modifications of skeletal muscle in modern broilers with a rapid growth rate can lead to myopathies such as white-striping (**WS**) (Livingston et al., 2019).

WS, a common myopathy, occurs primarily in heavier chicken with larger muscle fibers (Kuttappan et al., 2012; Brambila et al., 2016). Its pathology is characterized by the appearance of white striations running parallel to muscle fibers on the muscle surface, and is typically seen on breasts (Kuttappan et al., 2013a). This white striped feature on the muscle surface can negatively affect the visual appearance and consumer acceptability, since it can look like connective tissue or have a marbled appearance (Kuttappan et al., 2012). Thus, consumers tended to avoid purchasing chicken breast meat with WS (Kuttappan et al., 2012). Moreover, WS incidence could affect meat quality traits, especially water holding capacity (**WHC**). Petracci et al. (2013) reported a decreased WHC in chicken breast with WS, and thus resulting in tougher texture after cooking compared to normal chicken breast. Thus, more information is needed on how white striped feature affects the organoleptic characteristics of chicken meat, as these characteristics are important factors that influence the consumer acceptability and repurchase decision.

Sous-vide (**SV**) cooking has become one of the most preferred cooking methods because of its convenience and ability to extend the shelf-life of products (Bıyıklı et al., 2020). This method cooks vacuum-sealed raw foods in heat-stable pouches under precisely controlled temperatures (Baldwin, 2012; Kaur et al., 2020). It can make the sensory quality characteristics of various meat types more uniform and improved, especially tougher meat cuts, compared to the conventional cooking methods (Baldwin, 2012; Roldan et al., 2014; Hwang et al., 2019; Kim et al., 2019). Although this cooking method may improve the palatability of chicken breast meat, there is a lack of information about the effects of SV cooking condition on the sensory quality characteristics of chicken breast with WS. Therefore, this study was to compare the histochemical and meat quality characteristics between the normal and white striped chicken breasts. Additionally, this study also evaluated the effects of SV cooking on organoleptic characteristics of normal and white striped chicken breasts in order to improve the palatability and utilization of white striped chicken breast meat.

## MATERIALS AND METHODS

### Muscle Samples and Treatments

Broilers (2,169 ± 187.8 g) were slaughtered, and they were randomly selected and obtained three batches (10 chickens per batch) at the commercial slaughterhouse. Body weights (**BW**) of broiler chickens were individually measured before slaughter at the slaughterhouse. Both the left and right PM muscles from 30 broiler carcasses were obtained. A total of 30 left PM muscles were used for the measurements of histochemical and meat quality characteristics and a total of 30 right PM muscles were used for the evaluation of visual and eating quality traits. At 15 min postmortem, the left and right PM muscles were excised during standard slaughtering process of the Korea Institute for Animal Products Quality Evaluation (KAPE, 2020), and weighed in a 4°C cold room. The breast percentage was calculated as the proportion of the PM muscle weight (**PMW**) divided by the BW. Cross-sectional area (**CSA**) of PM muscle was measured according to previously published method (Scheuermann et al., 2004). Muscle pH was immediately measured on the left side of the muscle after obtaining PM muscles.

For evaluation of muscle fiber characteristics, samples were taken from the left PM muscle and cut into 0.5 × 0.5 × 1.0 cm pieces, then immediately frozen using liquid nitrogen and stored at −80°C. The remaining left and entire right PM muscles were immediately cooled using ice-cooled water, and stored at 4°C. After 24 h postmortem, the remaining left PM muscles were used to assess meat quality characteristics. The entire right breast muscles were classified according to their extent of WS (Kuttappan et al., 2013b). The normal group (n = 16) had no striping, and the WS group (n = 14) had moderate or severe striping. Trained panelists used the right PM muscles to evaluate the appearance of fresh meat. These muscles were then immediately frozen and stored at −20°C for the further treatments, including oven (**OV**) and SV cooking.

For the comparison of cooking loss, objective texture parameters, and sensory quality characteristics among the treatments, 15 PM muscles (8 normal; 7 WS) were roasted using the OV method (OV+Normal and OV+WS, respectively), and remaining 15 muscles (8 normal; 7 WS) were cooked using the SV method (SV+Normal and SV+WS, respectively). Electric oven (MJ324, LG Electronics, Seoul, Korea) was used for the OV method (set temperature, 180°C; end core temperature, 71°C; 10 min of mean cooking time per sample). For the SV groups, each PM muscle was packaged in a nylon-polyethylene pouches (200 × 300 mm; Rollpack, Pyeongtaek, Korea) and vacuum-sealed (Leepack, Hanguk Electronic, Incheon, Korea), then submerged in a temperature-controlled water bath (WSB-30, Daihan Scientific, Gangwon-do, Korea). Related study (Park et al., 2020) suggested the optimum condition for chicken breast was 60°C for 2 h, so we followed this accordingly.

### Histochemical Analysis

Using a cryostat (CM1510S, Leica, Nussloch, Germany) at −25°C, serial transverse muscle sections (10 μm) were obtained from each muscle sample. All muscle sections were stained using hematoxylin and eosin staining method (Cardiff et al., 2014). These stained slides were used to evaluate the muscle fiber characteristics, including average area and total number of muscle fibers. Each image was captured using an optical microscope (DM500, Leica Microsystems, Wetzlar, Germany) equipped with a high-definition digital camera (ICC50, Leica Microsystems, Milton Keynes, England) connected to a desktop computer with Leica software (LAS EZ, Heerbrugg, Switzerland). All histochemical images were examined using an image analysis system (Image-Pro Plus software, Media Cybernetics, Silver Spring, MD). At least 500 fibers per sample were evaluated for statistical analysis. The average CSA of muscle fibers was determined by dividing the total fiber area measured by the total fiber number. Total number of fibers was determined by multiplying muscle fiber density by CSA of PM muscle.

### Meat Quality and Visual Attributes of Fresh Meat

Muscle pH was measured on the surface of PM muscle at 15 min (pH_15 min_) and 24 h (pH_24 h_) postmortem using a pH instrument equipped with a penetration probe (Testo 206-pH2, Testo Inc., Lenzkirch, Germany). At 24 h postmortem, the meat color, including Commission Internationale de l'Eclairage (**CIE**) lightness (L*), redness (a*), and yellowness (b*) values, was measured at the upper surface region of a point 1/2 in the PM muscles using a Minolta chromameter (illuminant C; viewing angle, 0°; port/viewing area, 8 mm; CR-400, Minolta Camera Co., Osaka, Japan) according to the recommendations of CIE (1978). The meat color and drip loss were measured according to the procedures of Honikel (1998) and Kauffman et al. (1986). Before evaluations of visual attribute and sensory quality, 11 panelists (6 females and 5 males, ages 24 to 45) were trained with various sorts of commercial breast meat for a minimum of 6 mo (up to 1 h per session and 2 to 3 times per wk) (Meilgaard et al., 1991). All panel training and sensory evaluations were conducted at the Kyungpook National University. Panel training and sensory evaluations aligned with previous procedures (Meilgaard et al., 1991; American Meat Science Association, 2015; Lee et al., 2019). 9-point hedonic scale was used with end anchors of very unacceptable to very acceptable (1 to 9; low to high) to evaluate color, appearance, moisture, and overall acceptability of fresh breast meat.

### Cooking Loss and Objective Texture Parameters

Cooking loss and texture parameters were evaluated based upon methods outlined in a previous publication (Honikel, 1998). To assess the cooking loss, each sample was weighed before and after SV or OV cooking, and expressed as a percentage of cooking loss. SV and OV cooked samples were placed in a closed polyethylene bags and cooled in an ice-slurry, and then held at 4°C until Warner-Bratzler shear force (**WBS**) analysis. Samples (at least 6 cores; 1.27 cm diameter) were removed parallel to the muscle fiber orientation. WBS values were determined using an Intron Universal Testing Machine (Model 1011, Instron Corp., Canton, MA), the crosshead speed of the blade was operated 200 mm/min (American Meat Science Association, 2015). Cooked samples were cut into 2.0 × 2.0 × 1.5 cm, and then used for the texture profile analysis (TPA) using a TMS-touch texture analyzer (Food Technology Corp., Sterling, VA). The probe conditions and texture parameters were based on the method outlined by Bourne (1978).

### Sensory Evaluation of Cooked Meat

A total of 30 PM samples were randomly coded with a 3-digit number and evaluated during 6 sessions (5 samples for 1 section). After SV or OV cooking, cooked samples were trimmed into 1.3 cm^3^ cubes, and then kept in a sealed polyethylene bag and placed in a water bath at 52°C until served to the trained panelists. Prior to tasting each sample, panelists ate an unsalted cracker and rinsed their mouths with water. Ten attributes were evaluated including 6 tenderness attributes, juiciness, flavor intensity, off-flavor intensity, and overall acceptability (Park et al., 2020).

### Statistical Analysis

The general linear model was performed to elucidate any association for analyzing the effect of white striped features on histochemical, fresh meat quality, including visual attributes of fresh meat, and also determine the associations between the cooking methods and WS on the cooking loss, objective parameters, and palatability of cooked meat using SAS software (SAS Institute, Cary, NC). Cooking method, WS, and their interaction were employed as fixed effects in the model; panelists were employed as random effects. Significant differences in the least squares means (LSM) for investigated parameters between the treatments were compared using the probability difference, set at *P* < 0.05. All data were presented as LSM with standard errors.

## RESULTS

### Carcass Traits and Muscle Fiber Characteristics

Carcass traits and muscle fiber characteristics of the normal and WS groups are presented in Table 1. The normal group exhibited lower BW (2,090 vs. 2,266 g, *P* < 0.01), PMW (269 vs. 319 g, *P* < 0.001), and percentage of PMW relative to BW (12.8 vs. 14.1 %, *P* < 0.01) compared to the WS group. The WS group showed a greater muscle CSA compared to the normal group (22.0 vs. 19.1 cm^2^, *P* < 0.01). For the muscle fiber characteristics, broiler with a lower PMW exhibited a smaller area of muscle fiber compared to broiler with a heavier PMW (2,764 vs. 3,310 μm^2^, *P* < 0.01), whereas the total muscle fiber was similar between the groups (*P* > 0.05).

**Table 1:** Effects of white-striping on carcass traits and muscle fiber characteristics of *pectoralis major* muscle in broilers.

	Normal (N = 16)	White-striping (N = 14)	Level of significance
BW (g)	2090^b^ (40.8)^1^	2266^a^ (45.0)	⁎⁎
PMW (g)	269^b^ (9.82)	319^a^ (108)	⁎⁎⁎
PMW/BW (%)	12.8^b^ (0.30)	14.1^a^ (0.33)	⁎⁎
CSA of PM muscle (cm^2^)	19.1^b^ (0.61)	22.0^a^ (0.68)	⁎⁎
*Muscle fiber characteristics*			
Muscle fiber area (μm^2^)	2764^b^ (129)	3310^a^ (144)	⁎⁎
Total fiber number (× 1,000)	692 (37.3)	692 (33.7)	NS

Levels of significance: NS, not significant;

⁎⁎ *P* < 0.01.

⁎⁎⁎ *P* < 0.01.

a,b Different superscripts in the same row represent significant differences (*P* < 0.05).

1 Standard error of least square means. Abbreviations: BW, body weight; CSA, cross-sectional area; PMW, *pectoralis major* muscle weight.

### Meat Quality and Visual Attributes of Fresh Meat

Table 2 displays the meat quality and visual attributes for the normal and WS groups. With regard to characteristics of fresh meat quality, there were no significant differences between the normal and WS groups (*P* > 0.05). Marked differences were observed in visual attributes between the groups (*P* < 0.001) with the exception of moisture appearance (*P* > 0.05). The normal group did exhibit higher values of color (5.81 vs. 3.75, *P* < 0.001) and appearance (6.11 vs. 3.82, *P* < 0.001) acceptability compared to the WS group. Not surprisingly, overall acceptability was a higher score for the normal group compared to the WS group (5.89 vs. 3.75, *P* < 0.001).

**Table 2:** Effects of white-striping on fresh meat quality and visual attributes in broiler breasts.

	Normal	White-striping	Level of significance
*Muscle pH*			
Muscle pH_15 min_	6.54 (0.07)^1^	6.48 (0.07)	NS
Muscle pH_24 h_	5.64 (0.02)	5.61 (0.03)	NS
*Meat color*			
Lightness (L*)	55.1 (0.54)	55.6 (0.59)	NS
Redness (a*)	1.89 (0.21)	1.40 (0.23)	NS
Yellowness (b*)	4.86 (0.26)	5.01 (0.29)	NS
*Water holding capacity*			
Drip loss (%)	2.51 (0.21)	2.55 (0.23)	NS
*Sensory traits of fresh chicken breast*			
Color	5.81^a^ (0.14)	3.75^b^ (0.16)	⁎⁎⁎
Appearance	6.11^a^ (0.16)	3.82^b^ (0.18)	⁎⁎⁎
Appearance of moisture	6.13 (0.14)	5.71 (0.16)	NS
Overall acceptability	5.89^a^ (0.15)	3.75^b^ (0.16)	⁎⁎⁎

Levels of significance: NS, not significant;

⁎⁎⁎ *P* < 0.001.

a,b Different superscripts in the same row represent significant differences (*P* < 0.05).

1 Standard error of least square means.

### Cooking Loss and Textural Parameters of Cooked Meat

Combined effects of cooking treatment and white striped feature on cooking loss and textural parameters of broiler breasts are presented in Table 3. Chicken breast cooked by the OV method showed a higher cooking loss compared to breast cooked by the SV method (24.2 vs. 13.4 %, *P* < 0.05, data not shown), and a lower cooking loss was observed in the SV+WS group compared to the OV+Normal group (13.5 vs. 23.6 %, *P* < 0.05). There were no significant differences between the normal and WS groups within each cooking treatment for cooking loss and WBS (*P* > 0.05).

**Table 3:** Combined effects cooking methods and white-striping on cooking loss and objective texture properties in broiler breasts.

Cooking method (C)	Oven		Sous-vide		Level of significance		
White-striping (WS)	Normal(N = 8)	White-striping(N = 7)	Normal(N = 8)	White-striping(N = 7)	C	WS	C*WS
Cooking loss (%)	23.6^a^ (0.63)^1^	25.0^a^ (0.67)	13.3^b^ (0.63)	13.5^b^ (0.67)	⁎⁎⁎	NS	NS
WBS (N)	29.6^a^ (1.85)	32.0^a^ (1.97)	23.8^b^ (1.85)	21.1^b^ (1.97)	⁎⁎⁎	NS	NS
*Texture profile analysis*							
Hardness (N)	12.7^a^ (0.53)	12.5^a^ (0.57)	9.61^b^ (0.53)	8.80^b^ (0.57)	⁎⁎⁎	NS	NS
Adhesiveness (N·mm)	1.23^a^ (0.15)	0.13^b^ (0.16)	0.32^b^ (0.15)	0.24^b^ (0.16)	⁎⁎	⁎⁎⁎	⁎⁎
Cohesiveness	0.20^ab^ (0.01)	0.23^a^ (0.01)	0.17^b^ (0.01)	0.13^c^ (0.01)	⁎⁎⁎	NS	*
Springiness (mm)	4.51^b^ (0.42)	5.86^a’^ (0.45)	4.15^b^ (0.42)	3.92^b^ (0.45)	⁎⁎	NS	NS
Gumminess (N)	2.53^a^ (0.16)	2.82^a^ (0.17)	1.61^b^ (0.16)	1.15^b^ (0.17)	⁎⁎⁎	NS	*
Chewiness (N·mm)	11.7^b^ (1.55)	17.0^a^ (1.66)	6.80^c^ (1.55)	5.09^c^ (1.66)	⁎⁎⁎	NS	*

Levels of significance: NS, not significant;

⁎ *P* < 0.05;

⁎⁎ *P* < 0.01;

⁎⁎⁎ *P* < 0.001.

a,b,c Different superscripts in the same row represent significant differences (*P* < 0.05).

1 Standard error of least square means. Abbreviation: WBS, Warner-Bratzler shear force.

For the textural parameters, the WBS value was a higher in the OV group compared to the SV group (30.7 vs. 22.5 N, *P* < 0.05, data not shown). No difference was detected between the normal and WS groups within the OV or SV method (*P* > 0.05), whereas chicken breast from the OV+WS group showed a higher WBS value compared to chicken breast from the SV+WS group (32.0 vs. 21.1 N, *P* < 0.05). Similar to the WBS, TPA-hardness value did not differ between the normal and WS groups within the SV treatment (*P* > 0.05), and the OV+Normal group showed a higher value compared to the SV+Normal group (12.7 vs. 9.61 N, *P* < 0.05). Whereas, no significant differences were observed in the other TPA parameters between the normal and WS groups within the SV method (*P* > 0.05) except cohesiveness value (0.17 vs. 0.13, *P* < 0.05).

### Sensory Quality Characteristics of Cooked Meat

The interactions between cooking method and WS on sensory quality characteristics are presented in Table 4. Regardless of the presence of WS, PM cooked by the OV method showed lower values in all tenderness attributes compared to PM cooked by the SV method (*P* < 0.05). There were no significant differences in 6 tenderness attributes between the normal and WS groups within the SV method (*P* > 0.05). The OV+Normal group exhibited higher scores of softness (5.38 vs. 4.90, *P* < 0.05), amount of perceptible residue (5.27 vs. 4.38, *P* < 0.05), and overall acceptability (5.33 vs. 4.79, *P* < 0.05) than the OV+WS group. Significant difference was observed in juiciness between the normal and WS groups within the OV method (*P* < 0.05); however, there was no difference between the groups within the SV treatment (*P* > 0.05). Flavor intensity was a higher in the OV+WS group than the two groups within the SV treatment (6.24 vs. 5.78 and 5.39, *P* < 0.05), and lowest score of off-flavor intensity was observed for the SV+WS group compared to the other groups (*P* < 0.05). In addition, the OV+WS group exhibited a lower score of overall acceptability compared to the SV+Normal and SV+WS groups (4.93 vs. 6.64 and 5.91, *P* < 0.05).

**Table 4:** Combined effects of cooking methods and white-striping on sensory quality characteristics in broiler breasts.

Cooking method (C)	Oven		Sous-vide		Level of significance		
White-striping (WS)	Normal	White-striping	Normal	White-striping	C	WS	C*WS
*Tenderness attributes*							
Softness^2^	5.38^b^ (0.16)^1^	4.90^c^ (0.18)	7.38^a^ (0.16)	7.14^a^ (0.18	⁎⁎⁎	*	NS
Initial tenderness^3^	5.33^b^ (0.17)	4.91^b^ (0.19)	7.55^a^ (0.17)	7.09^a^ (0.19)	⁎⁎⁎	*	NS
Chewiness^4^	5.11^b^ (0.17)	4.72^b^ (0.19)	7.54^a^ (0.17)	7.17^a^ (0.19)	⁎⁎⁎	*	NS
Rate of breakdown^5^	5.60^b^ (0.16)	5.34^b^ (0.18)	6.97^a^ (0.16)	6.69^a^ (0.18)	⁎⁎⁎	NS	NS
Amount of perceptible residue^6^	5.27^b^ (0.23)	4.38^c^ (0.24)	6.18^a^ (0.23)	5.94^a^ (0.24)	⁎⁎⁎	*	NS
Overall tenderness^7^	5.33^b^ (0.15)	4.79^c^ (0.17)	7.26^a^ (0.15)	6.94^a^ (0.17)	⁎⁎⁎	*	NS
Juiciness^8^	4.76^b^ (0.16)	4.00^c^ (0.18)	5.87^a^ (0.16)	6.03^a^ (0.18)	⁎⁎⁎	NS	*
Flavor intensity^9^	5.82^ab^ (0.16)	6.24^a^ (0.17)	5.78^bc^ (0.16)	5.39^c^ (0.17)	⁎⁎	NS	*
Off-flavor intensity^10^	5.61^a^ (0.19)	6.09^a^ (0.21)	5.95^a^ (0.20)	4.73^b^ (0.20)	⁎⁎	NS	⁎⁎⁎
Overall acceptability^7^	5.07^c^ (0.18)	4.93^c^ (0.20)	6.64^a^ (0.18)	5.91^b^ (0.20)	⁎⁎⁎	*	NS

Levels of significance: NS, not significant;

⁎ *P* < 0.05;

⁎⁎ *P* < 0.01;

⁎⁎⁎ *P* < 0.001.

a,b,c Different superscripts in the same row represent significant differences (*P* < 0.05).

1 Standard error of least square means.

2 Scale: 1 = very hard, 9 = very soft.

3 Scale: 1 = very tough, 9 = very tender.

4 Scale: 1 = very chewy, 9 = very tender.

5 Scale: 1 = very slow, 9 = very fast.

6 Scale: 1 = very abundant, 9 = none.

7 Scale: 1 = dislike extremely, 9 = like extremely.

8 Scale: 1 = not juicy, 9 = extremely juicy.

9 Scale: 1 = very weak, 9 = very strong.

10 Scale: 1 = very strong, 9 = very weak.

## DISCUSSION

The incidence of myopathies, especially WS, has been steadily increasing in recent years, gaining considerable attention within the poultry industry (Petracci et al., 2019). Even though the etiology of the WS is still unknown, WS breasts had heavier and thicker than breasts with no striping (Mudalal et al., 2015). Our results were similar to those previous studies, as the WS condition broilers exhibited greater BW, PMW, and muscle CSA than the normal condition broilers (*P* < 0.001). Generally, ultimate muscle mass is dependent on the increase in the number of muscle fibers formed prenatally (fiber hyperplasia), as well as the increase in the size of these existing fibers during the postnatal period (fiber hypertrophy) (Picard et al., 2006; Choi et al., 2014). The rapid growth rate and greater muscle weight were observed in poultry line selected according to their BW and PMW compared to the random bred control line, which was due to fiber hypertrophy rather than hyperplasia (Scheuermann et al., 2004; Choi et al., 2013, 2014). In the current study, fiber area of broilers showing a heavier PMW of WS group was approximately 1.2 times greater than broilers showing a lighter PMW of normal group (*P* < 0.01), although no difference was observed in the total fiber number between the WS and normal groups (*P* > 0.05). Therefore, it can be inferred that the heavier and larger PM muscle of the WS broilers was mainly affected by muscle fiber hypertrophy rather than fiber hyperplasia.

The presence of WS can adversely affect the meat quality characteristics (Kuttappan et al., 2016). However, findings in the results on the relationship between WS and meat quality traits appear to conflict (Kuttappan et al., 2013a; Tasoniero et al., 2016). Alnahhas et al. (2016) remarked that breasts with severe WS were a lighter and exhibited a greater drip loss compared to normal breasts, although there were no differences between moderate and normal breasts. Kuttappan et al. (2013a) and Tasoniero et al. (2016) reported no significant differences in the meat quality traits between the normal and WS experimental groups. Similar result was observed in this study, no differences were observed in the fresh meat quality characteristics between the groups. Regarding consumer acceptance, Kuttappan et al. (2012) reported lower ratings for breasts with moderate and severe WS compared to breasts with no striping, and thus resulting in a higher score on “definitely would not buy” in purchase intent attributes. Thus, the WS appearance is an important factor influencing the purchase decision, as this stripe is considered as intramuscular fat from the consumer perspective (Petracci et al., 2019). In the present study, the trained panelists could distinguish the differences of visual appearance between the groups, and the WS group was assigned a lower score of overall acceptability than the normal group (*P* < 0.001).

SV method is characterized by low temperature long time (**LTLT**) processing under vacuum packaging, which minimizes the myofibrillar protein coagulation and water loss by evaporation compared to conventional cooking methods (Dominguez-Hernandez et al., 2018). Reduced cooking loss in SV treated meat is beneficial for the food industry, as cooking loss is associated with yield of the final products (Dominguez-Hernandez et al., 2018; Cho et al., 2020, Cho et al., 2021). As expected, the use of SV in our study minimized the cooking loss compared to the OV method (*P* < 0.001). Additionally, values for WBS and TPA-hardness were lower in the SV group compared to the OV group (*P* < 0.05), as the values of WBS and TPA-hardness are generally related to cooking loss and yield (Choi et al., 2019). However, there were no differences in values of cooking loss, WBS, and TPA-hardness depending on the presence of white striation (*P* > 0.05). Thus, the WS myopathy has had a somewhat limited effect on the variations of meat quality characteristics, including muscle pH, meat color, WHC, and textural properties, of chicken breast fillets.

Generally, meat cooked with LTLT is more tender than meat cooked with traditional cooking methods, as it minimizes the extent of fiber shrinkage (Dominguez-Hernandez et al., 2018). This result was associated with the differences of tenderness attributes between the SV and OV groups in this study. In the current study, less force was required to penetrate the SV sample during chewing than the OV sample, and panelists ranked overall tenderness higher in the breast fillets cooked by SV than the fillets cooked by OV (*P* < 0.05). Like the tenderness attributes, breast fillets from the SV treatment were juicier than breast fillets from the OV treatment (*P* < 0.05). Otherwise, severe WS breast fillets generally had a harder texture than normal fillets when cooked by conventional oven (Brambila et al., 2016). Within the OV treatment, WS breast fillets required a higher initial force of penetration, and had a greater amount of perceptible residue after chewing than normal fillets (*P* < 0.05). When chicken breast was cooked by the SV method, the trained panelists were not distinguishing the differences in all tenderness attributes between WS and normal fillets in this study (*P* > 0.05). Thus, SV cooking is a one of the best methods to improve the tenderness attributes of tough meat like WS breasts.

Meat contains a variety of flavor compounds that are formed or enhanced by thermal processing above 70°C (Dominguez-Hernandez et al., 2018; Ayub and Ahmad, 2019). The flavor generation of cooked meat also comes from the Maillard reaction, which a chemical reaction of free amino acids and reducing sugars that gives cooked meat its browned appearance (Dominguez-Hernandez et al., 2018). Park et al. (2020) reported that breast fillets cooked by OV at 180°C had greater flavor and off-flavor levels compared to fillets cooked by SV at 60 or 70 °C for 2 h due to lack of volatile aroma compounds from Milliard reaction, although showed a significantly lower score in overall tenderness acceptability compared to fillets cooked by SV. In the current study, relatively lower scores of flavor and off-flavor intensity were observed for PM cooked by SV at 60°C for 2 h than for PM cooked by OV (*P* < 0.05). On the other hand, the occurrence of abnormalities seen in fresh meat, especially white-striping and woody breast, can accelerate the lipid oxidation development that limit the shelf-life of chicken breast (Soglia et al., 2016). It is widely accepted that extent of lipid oxidation negatively influences palatability and consumer acceptance due to the increase of unacceptable flavor (Sampaio et al., 2012), and higher cooking temperatures tend to inhibit lipid oxidation (Byrune et al., 2002). Moreover, del Pulgar et al. (2012) reported that level of 2-thiobarbituric acid reactive substances associated with storage stability was decreased when SV cooking at 80°C compared to when SV cooking at 60°C. In the present study, within OV chicken fillets cooked at higher temperature, no significant difference was detected in the intensity of off-flavor between the WS and normal groups (*P* > 0.05). Whereas, panelists could detect more off-flavors in WS breasts than normal breasts when cooked by SV at 60°C for 2 h, demonstrating a higher overall acceptability for the SV+Normal group (*P* < 0.05).

## CONCLUSION

Taken together, increase in muscle fiber area was associated with an increased muscle mass and the occurrence of WS in chicken PM muscles. The meat quality characteristics did not differ between WS and normal breasts. After SV cooking at 60°C for 2 h, WS breasts required less penetrating force and were juicier compared to normal and WS breasts cooked by OV. WS fillets cooked by SV obtained a higher score of overall acceptability compared to normal fillets cooked by OV, although trained panelists preferred normal fillets cooked by SV due to less off-flavor. Therefore, the SV cooking can be an effective method for improving the palatability and utilization of WS chicken breast, and SV cooking could be combined with other cooking methods.

## DISCLOSURES

The authors did not provide a conflict of interest statement.
